# Efficacy and safety of eflornithine (CPP-1X)/sulindac combination therapy versus each as monotherapy in patients with familial adenomatous polyposis (FAP): design and rationale of a randomized, double-blind, Phase III trial

**DOI:** 10.1186/s12876-016-0494-4

**Published:** 2016-08-02

**Authors:** Carol A. Burke, Evelien Dekker, N. Jewel Samadder, Elena Stoffel, Alfred Cohen

**Affiliations:** 1 Department Gastroenterology & Hepatology, Cleveland Clinic, Mail Code A30, 9500 Euclid Ave, Cleveland, OH 44195 USA; 2 Department Gastroenterology & Hepatology, Academic Medical Center, C2-115, PO Box 22700, Amsterdam, 1100 DE The Netherlands; 3 Division of Gastroenterology, University of Utah, Huntsman Cancer Institute, 2000 Circle of Hope, Salt Lake City, UT 84112 USA; 4 Comprehensive Cancer Center, University of Michigan Health System, 1500 E. Medical Center Dr, Ann Arbor, MI 48109 USA; 5 Cancer Prevention Pharmaceuticals, Inc., 1760 E. River Rd, Tucson, AZ 85718 USA

**Keywords:** Chemoprevention, Colon polyps, Colorectal polyposis, Duodenal polyposis, Eflornithine, Familial adenomatous polyposis, Placebo controlled, Polyamines, Sulindac

## Abstract

**Background:**

Molecular studies suggest inhibition of colorectal mucosal polyamines (PAs) may be a promising approach to prevent colorectal cancer (CRC). Inhibition of ornithine decarboxylase (ODC) using low-dose eflornithine (DFMO, CPP-1X), combined with maximal PA export using low-dose sulindac, results in greatly reduced levels of normal mucosal PAs. In a clinical trial, this combination (compared with placebo) reduced the 3-year incidence of subsequent high-risk adenomas by >90 %. Familial Adenomatous Polyposis (FAP) is characterized by marked up-regulation of ODC in normal intestinal epithelial and adenoma tissue, and therefore PA reduction might be a potential strategy to control progression of FAP-related intestinal polyposis. CPP FAP-310, a randomized, double-blind, Phase III trial was designed to examine the safety and efficacy of sulindac and DFMO (alone or in combination) for preventing a clinically relevant FAP-related progression event in individuals with FAP.

**Methods:**

Eligible adults with FAP will be randomized to: CPP-1X 750 mg and sulindac 150 mg, CPP-1X placebo and sulindac 150 mg, or CPP-1X 750 mg and sulindac placebo once daily for 24 months. Patients will be stratified based on time-to-event prognosis into one of the three treatment arms: best (ie, longest time to first FAP-related event [rectal/pouch polyposis]), intermediate (duodenal polyposis) and worst (pre-colectomy). Stage-specific, “delayed time to” FAP-related events are the primary endpoints. Change in polyp burden (upper and/or lower intestine) is a key secondary endpoint.

**Discussion:**

The trial is ongoing. As of February 1, 2016, 214 individuals have been screened; 138 eligible subjects have been randomized to three treatment groups at 15 North American sites and 6 European sites. By disease strata, 26, 80 and 32 patients are included for assessment of polyp burden in the rectum/pouch, duodenal polyposis and pre-colectomy groups, respectively. Median age is 40 years; 59 % are men. The most common reasons for screening failure include minimal polyp burden (*n* = 22), withdrawal of consent (*n* = 9) and extensive polyposis requiring immediate surgical intervention (*n* = 9). Enrollment is ongoing.

**Trial registration:**

This trial is registered at ClinicalTrials.gov (NCT01483144; November 21, 2011) and the EU Clinical Trials Register(EudraCT 2012-000427-41; May 15, 2014).

**Electronic supplementary material:**

The online version of this article (doi:10.1186/s12876-016-0494-4) contains supplementary material, which is available to authorized users.

## Background

Familial adenomatous polyposis (FAP) is a rare, inherited gastrointestinal (GI) disorder caused by a mutation in the *Adenomatous Polyposis Coli* (*APC*) tumor-suppressor gene, located on chromosome 5q21-22 [1]. FAP is characterized by the early onset, usually in adolescence, and gradual development of hundreds to thousands of adenomatous polyps in the colon, rectum and duodenum [2]. If left untreated there is a nearly 100 % risk of colorectal with an average age of diagnosis of 39 years [3–5].

Prophylactic surgery in young adults, including total colectomy with ileo -rectal anastomosis (IRA), is the mainstay of treatment and mitigates the development of colorectal cancer in most patients. Individuals with extensive rectal involvement usually undergo total proctocolectomy with ileal-pouch anal anastomosis (IPAA) [3]. Many such patients develop progressive polyposis in the retained rectum [6, 7] or ileal pouch [8–10]. Despite excision of the main at-risk organ(s), many patients develop duodenal adenomas and require frequent surveillance and endoscopic or surgical intervention [11]. This is particularly problematic in patients with mesenteric desmoids disease. After colectomy, the main causes of morbidity and mortality in FAP are duodenal cancer and desmoid disease. Thus, there is an ongoing unmet need for an effective chemo-preventive option.

**Fig. 1:**
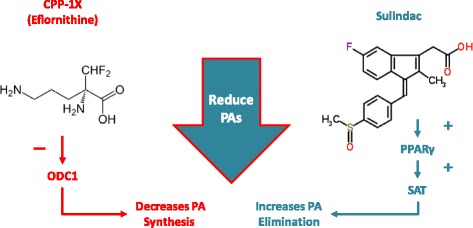
CPP-1/sulindac downregulates PAs via dual MoA: CPP-1X decreases PA synthesis by blocking ODC1, and sulindac increases PA catabolism and export by upregulating transport genes (PPARγ and SAT). MoA, mechanism of action; ODC1, ornithine decarboxylase; PA, polyamine; PPAR, peroxisome proliferator activated receptor; SAT, sialic acid transport

The *APC* gene suppresses the transcription of several oncogenes, including *MYC*, which in turn, regulates the expression of ornithine decarboxylase (ODC). In patients with FAP, ODC enzyme activity and polyamine (PA) levels are markedly increased in the normal colonic mucosa relative to genotype negative family members [12]. Therefore, PA reduction might be a potential strategy to control the progression of FAP-related colorectal polyposis, and findings from molecular studies provide support for this hypothesis [13] (Fig. 1). In a mouse model of FAP, the combination of eflornithine (difluoromethylornithine [DFMO], CPP-1X) and sulindac or celecoxib non-steroidal anti-inflammatory drugs (NSAIDs) led to marked reductions in intestinal tumors compared to each agent alone. Only the DFMO and sulindac combination reduced total intestinal PA contents [14].

This Phase III, 2-year treatment trial is designed to evaluate the efficacy of a novel combination therapy in patients with FAP using clinically relevant outcomes such as delay in FAP-related excisional intervention involving the colon, rectum pouch and/or duodenum or delay in progression to more advanced duodenal polyposis, cancer or death.

## Methods/Design

### Study objectives

The primary objective of this study is to determine whether CPP-1X/sulindac combination therapy is superior to either given as monotherapy for 24 months among patients with FAP. Clinical benefit is defined as a delay in the time to the first FAP-related disease progression event with combination therapy versus monotherapy.

### Study design

CPP FAP-310 is an ongoing, randomized, double-blind, active-controlled, Phase III trial evaluating the efficacy and safety of CPP-1X/sulindac combination therapy versus each as monotherapy during a 24-month treatment period among individuals with FAP [15]. Patients are assessed endoscopically every 6 months for evidence of polyposis progression (vide infra).

### Participating centers

CPP FAP-310 is being conducted at 15 sites in North America and 6 sites in Europe. North American sites include the Cleveland Clinic in Ohio and Florida, University of Utah, Mount Sinai Hospital and University Health Network in Ontario, Canada, MD Anderson Cancer Center in Texas, Dana Farber Cancer Institute in Massachusetts, Mayo Clinic in Minnesota, University of Michigan, Washington University School of Medicine, University of Pennsylvania, University of Washington, University of California San Diego, Emory University in Georgia, University of Wisconsin-Madison and Vanderbilt University in Tennessee.

European sites include the Academic Medical Centre in The Netherlands; University of Bonn Hospital in Germany; Institut de Malalties Digestive in Spain; Institute of Genetic Medicine, Newcastle and the Manchester Centre for Genomic Medicine, University of Manchester in the United Kingdom and Leuven Cancer Institute in Belgium.

### Study population

**Fig. 2:**
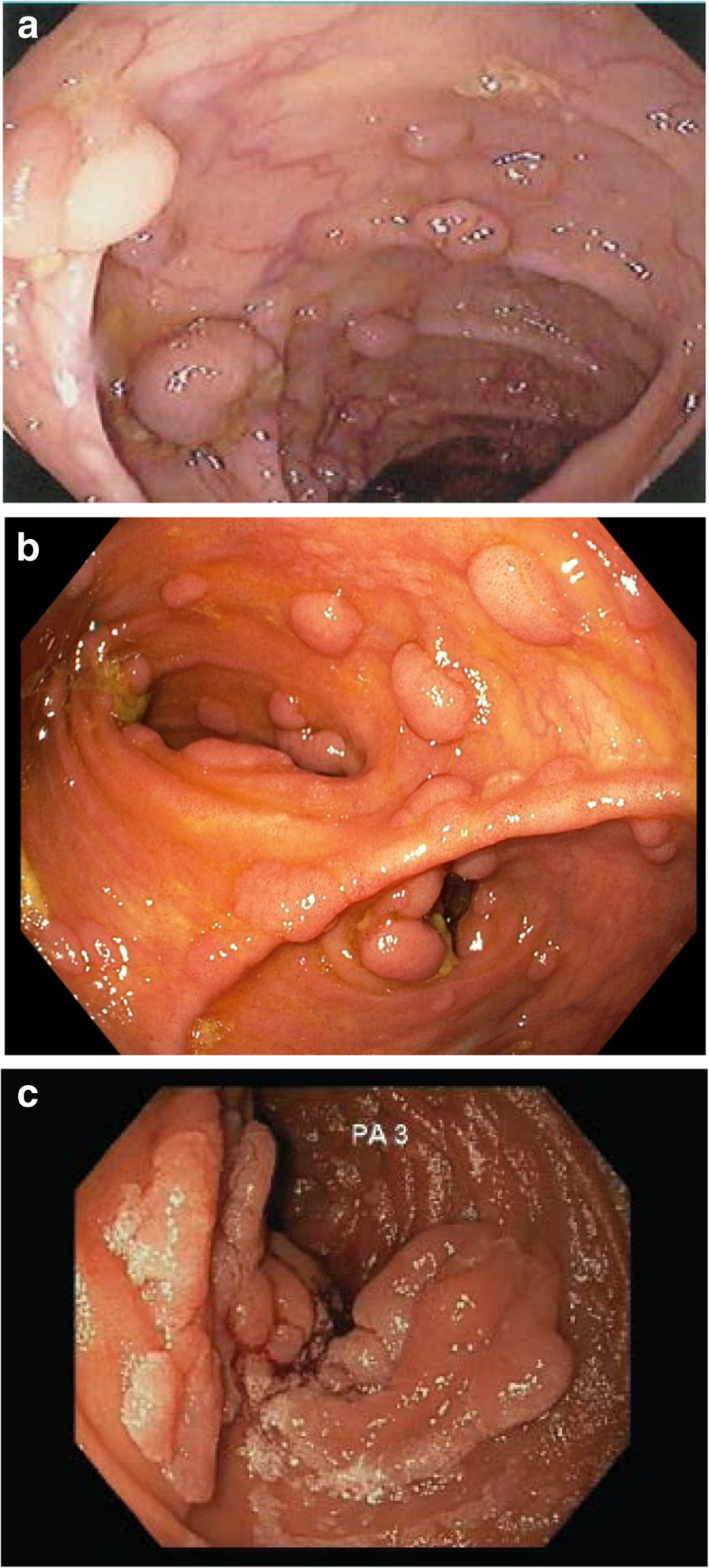
Endoscopic images of patient strata of polyposis: **a** pre-colectomy, **b** rectal/pouch, and **c** duodenal (Spigelman stage 3/4)

Eligible participants (aged ≥18 y) must have a documented, genotyped *adenomatous polyposis coli* (*APC*) mutation associated with the classic FAP phenotype, which is typically characterized by an autosomal dominant pattern of inheritance, onset in adolescence, >100 colorectal adenomas, and 100 % risk for CRC [5]. This includes participants with intact colons/rectums being considered for prophylactic surgery, those with IRA or IPAA International Society for Gastrointestinal Hereditary Tumours [InSiGHT] stage 1, 2 or 3 polyposis in the retained rectum or pouch ≥3 years before enrollment, and participants with duodenal polyposis, Spigelman Stage 3 or 4 (Table 1; Fig. 2). The modified version of the Spigelman Staging System is provided in Table 2.

**Table 1:** Patient stratification: eligibility criteria

Patient stratum	Description of criteria
Pre-colectomy (Fig. 2a)	• Patients with an intact colon/rectum considering prophylactic surgery
Retained rectum/ileal pouch polyposis (Fig. 2b)	• Patients with ≥3 years since colectomy with IRA/proctocolectomy with pouch and demonstrating polyposis as defined by Stage 1, 2, 3^a^: − Stage 1: 10–25 polyps, all <5 mm − Stage 2: 10–25 polyps, at least one >1 cm − Stage 3: >25 polyps amenable to complete removal, or any incompletely removed sessile polyp, or any previous evidence of high-grade dysplasia, even if completely removed • For all patients, any rectal/pouch polyps >5 mm must be excised at baseline
Duodenal polyposis (Fig. 2c)	• Patients with ≥1 of the following: − Current Spigelman Stage 3 or 4 − Patients who had previous surgical endoscopic intervention ≤6 months for Spigelman Stage 3/4 that may have been down-staged to Spigelman 1/2

^a^InSiGHT 2011 Staging System (InSiGHT Meeting, 2011, San Antonio, TX)

**Table 2:** Modified Spigelman’s Score and Classification [26]

	Score				
Factor	1 Point	2 Points	3 Points	Total Score	Stage
Number of polyps	1–4	5–20	>20	0	0
Polyp size, mm	1–4	5–10	>10	1–4	1
Histology	Tubulous	Tubulovillous	Villous	5–6	2
Dysplasia	Low grade	—	High grade^a^	7–8	3
				9–12	4

^a^Assigned to any epithelium showing nuclear stratification all the way to the tops of the cells and loss of mucin production. It can encompass intraepithelial carcinoma if the cells are pleomorphic or even cribiformed but still all located above the basement membrane

Key exclusion criteria include significant cardiovascular risk factors (uncontrolled high blood pressure, unstable angina, history of myocardial infarction, uncontrolled hyperlipidemia) and clinically significant hearing loss requiring the use of a hearing aid. A full listing of the inclusion and exclusion criteria is provided in the Additional file 1.

### Primary outcome measure

The primary efficacy endpoint is the time to first occurrence of any FAP-related event in the patient as a whole (more than one site may be at risk). This includes: (1) FAP-related excisional intervention involving the colon, rectum, pouch, duodenum and/or (2) clinically important events that include progression to more advanced duodenal polyposis (Stage 2–4), cancer, or death. Excisional interventions include surgery or advanced endoscopic procedures. Definitions for FAP-related disease progression by patient stratum are provided in Table 3.

**Table 3:** Definition of FAP-related disease progression by patient stratum

Patient stratum	Disease progression
Pre-colectomy	• ≥25 % increase in polyp burden (number, size) from baseline • Presence of large, sessile or ulcerated adenoma (not amenable to excision) • High-grade dysplasia • Large villous adenoma • In-situ or invasive cancer
Retained rectum/ileal pouch polyposis	• ≥25 % increase in polyp burden (number, size) from baseline • Excisional intervention to remove any polyp ≥10 mm • High-grade dysplasia in any polyp • In-situ or invasive cancer on any biopsy
Duodenal polyposis	• Increase in Spigelman Stage (2–4) from baseline • Need for excisional intervention • Development of cancer • Death (endoscopy/intervention related)

### Secondary outcome measures

Secondary endpoints include the evaluation of potential effect-modifying properties of the presence or absence of an ODC polymorphism and the pharmacokinetics, tissue and dietary levels, and urinary excretion of PAs. Treatment effects on health-related quality of life (HRQoL) will be assessed using the patient-reported EORTC core questionnaire (QLQ C30) [16], GI-specific module of EORTC questionnaire (QLQ CR29) [17], the EuroQol EQ-5D [18, 19] and a modified version of the Cancer Worry Scale [20]. These instruments will be administered at baseline and at 3, 6, 12, 18 and 24 months post-enrollment/end of treatment.

### Treatment, procedures and assessments

A total of 150 eligible patients will be randomized (1:1:1) to one of three treatment groups: (1) CPP-1X 750 mg with sulindac 150 mg, (2) CPP-1X placebo with sulindac 150 mg, and (3) CPP-1X 750 mg with sulindac placebo. Study drugs are taken orally, once daily as four tablets (three 250-mg CPP-1X/CPP-1X placebo tablets and one 150-mg sulindac/sulindac placebo tablet). For all randomized patients, treatment will continue for 24 months, or until the occurrence of an FAP-related event, as defined in Table 3.

Patients will undergo upper and lower GI endoscopy every 6 months using standard sedation according to local clinical management procedures. Endoscopies are videotaped, with key still photographs, which are de-identified and stored for subsequent review. A forward-viewing endoscope will be used to perform three spiral passes from the third portion of the duodenum to the duodenal bulb including a side-viewing duodenoscopy to visualize the papilla, and from the top of the ileal pouch or ileo-rectal anastomosis to the anal verge. In patients with an intact colon, a single spiral pass from the cecum to the anal verge will be performed. All videos include a standard 2.4-mm endoscopic biopsy forceps in the field of view. Baseline biopsies are obtained for duodenum staging (Spigelman scoring), and in the rectum, pouch or colon, biopsies are obtained for research purposes and for the removal of concerning adenomas. Medications and patient diaries are provided to participants every 3 months. In the diaries, patients are instructed to record medication use, presence of symptoms, and self-assessment of bleeding or melena. A detailed schedule of study visits and follow-up is included in the study protocol and clinical trial registry records [15].

### Safety and tolerability

Adverse events (AEs) and adverse drug reactions (ADRs) will be monitored. AEs are defined as any untoward medical occurrence regardless of its relationship to study medication. Serious adverse events (SAEs) include death, a life-threatening event, hospitalization or prolonged hospitalization, persistent or significant disability, congenital abnormality/birth defect, and any event that may jeopardize the patient’s well-being. ADRs are events judged to be related to study medication. AE and SAE reporting and grading will be performed using the National Cancer Institute’s Common Terminology Criteria for Adverse Events (CTCAE) Version 4.0 [21]. Laboratory results, including hematology, chemistry and urinalysis, will be compared over time (ie, screening; months 3, 6, 12 and 18; and end of treatment) to detect any safety signals. Patients will be followed for safety from start of treatment through 30 days after treatment discontinuation. Serious adverse events (SAEs) will be followed until they are resolved or return to baseline values.

### Adverse events of special interest

To assess cardiac risk, patients will undergo ECG evaluation at baseline and at months 3, 6, 12, 18 and 24. To assess ototoxicity risk, all patients will undergo air conduction audiometry at screening and at months 12 and 24. To assess gastrointestinal risk, patients will conduct stool assessments and record the findings in their diaries.

### Statistical analysis

CPP FAP-310 is a superiority trial, designed to detect a delay in the primary efficacy endpoint in favor of CPP-1X and sulindac combination therapy compared with either as monotherapy.

#### Sample size

Sample size determination was based on limited data from CPP-1X and sulindac monotherapy studies in which the 2-year event-free rates implied a single, overall event-free rate of 60 % for the combination therapy group and 30 % for each monotherapy group. Several assumptions have been made. Statistical analysis will involve two-sided log-rank tests (α = 0.05) for the time to first FAP-related event for two between-group comparisons: CPP-1X with sulindac versus CPP-1X and CPP-1X with sulindac versus sulindac. The time to the first FAP-related event for either monotherapy group is expected to be approximately twice that for the combination therapy group. There will be ≥85 % power to detect an effect of treatment when comparing combination therapy with either as monotherapy. Finally, the two monotherapy groups will have the same event rate.

With 50 patients per treatment group and assuming 2-year time-to-first-FAP-related event rates of 70 % for the monotherapy groups and 40 % for the combination therapy group, the expected number of patients with an FAP-related event would be 35 in each of the monotherapy groups and 20 in the combination therapy group. For the between-group comparisons, it would be expected that 55 patients (SD 4.74) would have an FAP-related event, corresponding to nearly 89 % power. A doubling in event-free follow-up over 2 years corresponds to the design effect size, a hazard rate ratio of 0.4243 (ln 0.60)/(ln 0.30).

#### Data analysis

The primary efficacy analysis will be conducted on the intent-to-treat (ITT) population, defined as all patients randomized to study treatment; safety assessments will be performed on the safety population, which includes all randomized patients who received ≥1 dose of study drug. Baseline demographic characteristics will include age, gender, and race; clinical characteristics will include disease-related features and laboratory values. Continuous variables will be summarized as means (with SDs) or medians (with ranges), and categorical variables will be presented as frequency distributions and percentages.

For the primary, time-to-event analysis, a stratified log-rank test will be used, and data will be displayed using the Kaplan-Meier method. If an FAP-related event occurs, that patient will be recorded as having an observed or uncensored event and will be considered a treatment “failure.” Cox proportional hazard regression models will be used for secondary analyses.

For the between-treatment group comparisons, continuous data will be analyzed using an analysis of covariance, with baseline value, a binary indicator variable for the two highest-risk patient strata, and a binary indicator variable for treatment (combination versus monotherapy) as covariates. Categorical data will be analyzed using chi-square tests and Cochran-Mantel Haenszel tests to reflect the stratified randomization. Ordered categorical data will be analyzed using Kruskal-Wallis nonparametric tests.

For the EORTC QLQ-C30 and QLQ-CR29, each single item or multi-item subscale scores will be standardized to a scale of 0–100 by applying a linear transformation. For HRQoL secondary endpoints, single- or multi-item subscale scores will be categorized as improved or deteriorated if the change from baseline is ≥10 points. For the EuroQoL EQ-5D, patient preferences (or utilities) will be assessed by determining preference weights among the treatment groups for individual health states, and quality-adjusted survival will be generated by multiplying the utility value by the amount of time spent in a specified health state. The modified version of the Cancer Worry Scale will be administered and scored as previously described [20].

### Baseline demographic and clinical characteristics (interim results)

**Fig. 3:**
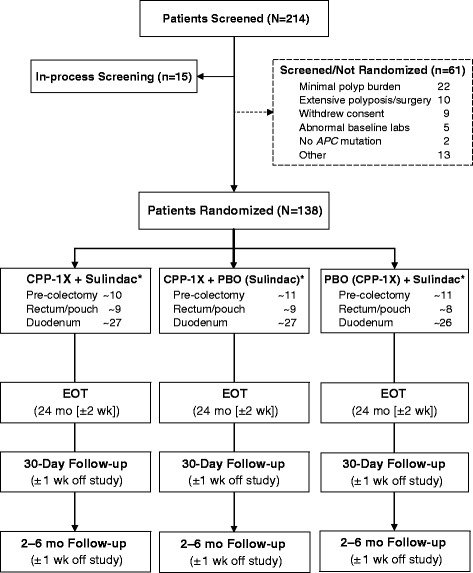
Flowchart of the study (as of February 1, 2016). *Treatment arm is assigned in double-blinded fashion; exact numbers of randomized patients per treatment arm are not known. APC, adenomatous polyposis coli; CPP-1X, eflornithine; EOT, end of treatment; PBO, placebo

As of February 1, 2016, 214 individuals have been screened, and 138 eligible subjects have been randomized to one of the three treatment groups (Fig. 3). The randomized population has a median age of 40 years and includes 81 male and 57 female subjects. The enrollment period to date is 24 months. The most frequent reasons for screen failure include minimal polyp burden (*n* = 22), extensive polyposis requiring immediate surgical intervention (10), withdrawal of consent (*n* = 9), abnormal baseline labs (*n* = 5), and no *APC* mutation (*n* = 2).

To date, 8 SAEs have been reported. Worsening of depression with suicidal ideation (*n* = 1) and deep vein thrombosis (*n* = 1) have been assessed as being possibly related to study treatment; severe seasonal migraine (*n* = 1), post-polypectomy bleed (*n* = 1), adhesive small bowel obstruction (*n* = 1), lung adenocarcinoma (*n* = 1), small bowel ileus (*n* = 1) and pancreatitis (*n* = 1) have been assessed as not being related to study treatment. All subjects experiencing an SAE were stratified to the duodenal polyposis group.

## Discussion

FAP is a rare disease (1/10,000) with multiple, major unmet medical needs. The current initial standard of care is prophylactic colectomy or proctocolectomy, followed by regular and lifelong endoscopic evaluation, polypectomies or laser/cautery ablation and additional operations. Controlling the progression of colorectal polyposis burden, minimizing the development of high-grade dysplasia and avoiding interval cancer would provide patients and physicians with the ability to prevent or delay prophylactic surgery in younger patients to a more convenient time. Specific control of rectal polyposis may increase the likelihood that a colectomy with IRA can be performed as the initial procedure. Bowel function, anal incontinence and female fecundity may be improved without the pelvic dissection and ileal pouch required for proctocolectomy.

Approximately 50 % of patients with retained rectum or ileal pouch develop adenomatous disease requiring frequent endoscopies, polypectomies and ablation. Although cancer is infrequent, regular proctoscopies are inconvenient and increased post-polypectomy scarring can result in loss of compliance, increased stool frequency and increased fecal urgency.

Duodenal polyposis occurs in >90 % of patients and requires regular surveillance. Both advanced or symptomatic ampullary disease and large duodenal polyps require complex endoscopic and surgical procedures even in the absence of invasive cancer. This problem represents the major source of morbidity and premature mortality in FAP patients.

The CPP FAP-310 trial combines patients with all three major life-time FAP-related polyposis disease sites: (1) primary colorectal, (2) retained rectum/ileal pouch, and (3) advanced duodenal disease. The major objective of this clinical trial program is to defer or obviate the need for additional surgical interventions in FAP patients. Secondarily, the study will determine treatment effects on HRQoL.

The development of novel pharmacotherapies that effectively increase the time to FAP-related events are important for controlling the morbidity and mortality associated with this genetic disease. Sulindac has been used “off-label,” particularly to control colorectal polyposis. However, sulindac monotherapy for the treatment of duodenal polyposis has shown little to no efficacy [22, 23]. Celecoxib was approved by the FDA in 1999 as an adjunct to endoscopy to treat FAP based on data from a 6-month trial demonstrating a 28 % reduction in polyp count in a defined segment of the bowel [24]. However, because regulatory post-market clinical studies were not completed, and evidence of clinical benefit was not provided, the indication for FAP was removed from celecoxib’s product label.

In a study by Lynch and colleagues, the relative efficacy of combination therapy with DFMO and celecoxib versus celecoxib monotherapy was evaluated in FAP patients [25]. In this trial, 112 patients were randomized, but only 68 patients were evaluable at 6 months (ie, baseline and 6-month endoscopy data for those who took most of their daily drug dosage by this time point). The study used three key endpoints. The primary endpoint was polyp counts in designated areas (“defined anatomic fields”) of the bowel. Secondary endpoints focused on changes in polyp burden (number and size) as determined using still images and video assessments. In the still images, the number and size of polyps were estimated in the “defined anatomic fields.” Open and closed forceps were used for size estimation. In the video assessment, five independent reviewers evaluated four segments of the colon and rectum, or if post-colectomy, only the rectum. Each segment was evaluated at 6 months and compared with baseline. Grades included: much better, better, the same, worse or much worse.

The primary endpoint data trend supported the benefit of the combination therapy, but because of the relatively small sample size, the difference was not statistically significant. When comparing polyp counts or burden in pre-defined areas of still photos, DFMO and celecoxib combination therapy was not superior to celecoxib monotherapy. However, when the polyp burden was assessed for the entire colorectum by multiple reviewers of blinded video-endoscopy, the DFMO/celecoxib group showed 93 % improvement and the celecoxib monotherapy group showed 36 % improvement (*p* = 0.01). Clinical care and management decisions focus on the entire bowel polyp burden as well on high-risk adenomas (large and/or with high grade dysplasia). No data were presented with regard to high-grade dysplasia.

The Lynch report provided additional support for the proof-of-principle concept for the current trial; however, there are important differences in the trial designs. First, the previous study was short (ie, 6 months) and included a relatively small number of patients (ie, 68). By contrast, in the CPP FAP-310 trial, patients are receiving CPP-1X and sulindac combination therapy for 24 months, which is critical for determining clinically relevant outcomes. The target sample size is 150 patients, and nearly 95 % of this total is currently enrolled. In the previous study, the primary endpoint of counting polyps in small, designated areas of the colon is not clinically meaningful. The secondary endpoint of changes in global polyp burden is more predictive of clinical benefit.

In the celecoxib registration trial, patients received 200 mg/day or 800 mg/day celecoxib monotherapy, much higher than the standard NSAID dose. Two patients in the 800-mg group withdrew from the trial owing to an allergic reaction and dyspepsia [24]. In the Lynch report, patients received 800 mg/day celecoxib. In the celecoxib and placebo group, 20 % (11/55) of patients reported fatigue, 20 % (11/55) experienced mucositis/stomatitis; 10 % (6/55) experienced diarrhea and 10 % (6/55) had nausea/vomiting [25]. In the CPP FAP-310 study, the dose of sulindac is one-half that of a standard NSAID dose. For safety reasons, the use of low-dose sulindac is preferred, as the drug regimen is likely to be used for many years by patients with FAP.

For the CPP FAP-310 trial, the efficacy endpoints are based on specific input received from regulatory agencies in the United States and Europe and are focused on providing “clinically meaningful” results. That is, findings for which the effects of treatment are large and perceived to be of benefit from the patient’s perspective. Indeed, avoiding or delaying major excisional interventions as defined in the FAP-310 trial is clearly meaningful to patients.

In general, the clinical management of FAP is driven by the progression of polyp burden, resulting in “FAP-related events,” as specified by these regulatory agencies. By extension, polyp burden regression is associated with delaying progression and mitigating FAP-related events. A delay in the time to colectomy or proctocolectomy is important to patients. Avoiding major resections of the duodenum greatly reduces morbidity and mortality. Preserving bowel function in the retained rectum or ileal pouch by reducing the need for repeated excisional interventions is an additional unmet medical need. All of these will be evaluated in the CPP FAP-310 trial, setting a precedent for the inclusion of more rigorous, clinically relevant efficacy assessments for this and future FAP trials.

## Abbreviations

ADR, adverse drug reaction; AE, adverse event; ANCOVA, analysis of covariance; *APC*, *adenomatous polyposis coli* tumor-suppressor gene; BID, twice daily; CI, confidence interval; COX, cyclooxygenase; CPP-1X, eflornithine; DFMO, difluoromethylornithine; EORTC QLQ-C30, European Organization for Research and Treatment Center Quality of Life Questionnaire – Core; EORTC QLQ-CR29, European Organization for Research and Treatment Center Quality of Life Questionnaire – Colorectal Cancer Module; FAP, familial adenomatous polyposis; GI, gastrointestinal; HRQoL, health-related quality of life; IPAA, ileal-pouch anal anastomosis, InSiGHT, International Society for Gastrointestinal Hereditary Tumors; IRA, ileo-rectal anastomosis; ITT, intent-to-treat population; NSAID, non-steroidal anti-inflammatory drug; ODC, ornithine decarboxylase; QD, once daily; SAE, serious adverse event; SD, standard deviation

## Additional file

Additional file 1: Study protocol inclusion and exclusion criteria. (DOCX 17 kb)
