# Supplementary material for: Efficacy and safety of eflornithine (CPP-1X)/sulindac combination therapy versus each as monotherapy in patients with familial adenomatous polyposis (FAP): design and rationale of a randomized, double-blind, Phase III trial
Source: BMC Gastroenterol. 2016 Aug 2;16:87. doi: 10.1186/s12876-016-0494-4 (PMC4969736; doi:10.1186/s12876-016-0494-4)
Supplement: Additional file 1: — Study protocol inclusion and exclusion criteria. (DOCX 17 kb) [file 12876_2016_494_MOESM1_ESM.docx]

**Additional file**

**Additional file 1: Study protocol inclusion and exclusion criteria**

- Diagnosis of phenotypic classical FAP with disease involvement of the duodenum and/or colon/rectum/pouch
  - Genotype: APC mutation (with or without family history) required
  - Classical FAP Phenotype: 100s to 1000s of colorectal adenomatous polyps, usually appearing in teenage years
- Upper/lower GI endoscopy performed ≤30 days of randomization
- Hematopoietic Status (≤30 days of randomization)
  - No significant hematologic abnormalities
  - White blood cell count ≥3,000/mm^3^
  - Platelet count ≥100,000/mm^3^
  - Hemoglobin ≥10.0 g/dL
  - No history of clinical coagulopathy
- Hepatic Status (≤30 days of randomization)
  - Bilirubin ≤1.5 times ULN
  - AST and ALT ≤1.5 times ULN
  - Alkaline phosphatase ≤1.5 times ULN
- Renal Status (≤30 days of randomization): creatinine ≤1.5 times ULN
- Hearing: no clinically significant hearing loss
- Female patients: neither pregnant nor lactating; negative pregnancy test; fertile patients required to use effective contraception
- Absence of gross blood in stool; red blood on toilet paper only acceptable
- No discrete gastric or duodenal ulcer >5 mm in past year except *Helicobacter pylori*-related peptic ulcer disease treated with antibiotics
- No invasive malignancy ≤5 years except resected non-melanomatous skin cancer, papillary thyroid cancer or precancerous cervical dysplasia
- No other significant medical or psychiatric problems that would preclude study participation or interfere with capacity to give informed consent
- Daily use of 81–100 mg or weekly use of ≤700 mg aspirin allowed
- No concurrent warfarin, fluconazole, lithium, dabigatran or other direct thrombin inhibitors, clopidogrel, cyclosporine, other NSAIDs (eg, ibuprofen, aspirin [>700 mg/wk], diflunisal), diuretics (furosemide and thiazides), DMSO, methotrexate, probenecid, propoxyphene hydrochloride, acetaminophen, preparations containing aspirin or cytotoxic chemotherapy drugs
- Willingness to forego concurrent use of supplements containing omega-3 fatty acids, oral corticosteroids, non-steroidal anti-inflammatory drugs or other FAP-directed drug therapy
- Able to provide written informed consent and follow protocol requirements

***Exclusion Criteria***

- Previous pelvic irradiation
- Patients receiving oral corticosteroids ≤30 days of enrollment
- Treatment with other investigational agents in the previous 4 weeks
- Use of other NSAIDS (eg, ibuprofen) >4 days/month, in the previous 6 weeks
- Regular use of aspirin in >700 mg/week
- Treatment with other FAP-directed drug therapy (including sulindac, celecoxib, fish oil) ≤12 weeks of enrollment
- Hypersensitivity to cyclooxygenase-2 inhibitors, sulfonamides, NSAIDs or salicylates; NSAID-associated symptoms of gastritis
- Patients must not have cardiovascular disease risk factors as defined below:
- Uncontrolled high blood pressure (systolic blood pressure > 150 mm Hg
- Unstable angina
- History of documented myocardial infarction or cerebrovascular accident
- New York Heart Association Class III or IV heart failure (Refer to Appendix C)
- Known uncontrolled hyperlipidemia defined as LDL-C ≥ 190 mg/dL or triglycerides ≥500 mg/dL
- Patients with significant hearing loss that affects everyday life and/or for which a hearing aid is required
- Intact colon/rectum or retained rectum or ileal pouch:
- Cancer on biopsy
- High-grade dysplasia on polyp biopsy, where the polyp is not completely removed
- Large polyp (>1 cm) not completely removed
- Duodenal cancer on biopsy
- Intra-abdominal desmoid disease stage III or IV
- Inability to provide informed consent
